# Supplementary material for: Are carbon emissions trading and green financial instruments synergistic? -Comprehensive quantitative research based on content analysis
Source: PLoS One. 2024 Mar 7;19(3):e0298601. doi: 10.1371/journal.pone.0298601 (PMC10919601; doi:10.1371/journal.pone.0298601)
Supplement: S1 Data — (ZIP) [file pone.0298601.s001.zip › Data/地级市Z_F工作报告.zip!/║ú─╧/║ú┐┌╩╨/└┤╫╘╣1⁄2╓┌║┼í╛┬φ┐╦╩2╛▌═°í┐_2009.docx]

各位代表：

现在，我代表市人民政府向大会报告工作，请予审议，并请市政协各位委员和其他列席人员提出意见。

一、2008年工作回顾

2008年是海口发展进程中极不寻常的一年。我们圆满完成建省办经济特区20周年庆典任务，精心组织世界关注的奥运火炬传递活动，全力支援四川汶川地震灾区，积极抗击特大洪涝灾害。特别是面对国际金融危机对经济发展的影响，全市人民在省委、省政府和市委的坚强领导下，深入贯彻落实党的十七大、胡锦涛总书记视察海南重要讲话、省委五届三次、四次全会和市委十一届三次、四次全会精神，抢抓机遇，奋力拼搏，全市经济继续保持平稳较快增长势头。

经济实力进一步提升。全市实现生产总值443.2亿元，增长10.4%;财政总收入100.02亿元、增长21.2%，地方一般预算收入31.03亿元、增长19.1%。

投资与消费进一步增长。全社会固定资产投资完成219.1亿元，增长20.5%;社会消费品零售总额达234.8亿元，增长24%。投资和消费拉动GDP增长8.94个百分点。

人民生活进一步改善。城镇新增就业3.1万人，农村劳动力转移就业11290人。物价自去年5月逐月回落，12月份CPI已降至2.2%。城镇居民人均可支配收入达到14150元，农民人均纯收入达到5215元，分别增长15.1%和13.8%，城乡居民收入增幅再创新高。

一年来，我们主要做了以下八个方面的工作：

(一)沉着应对各种挑战，积极抢抓发展机遇。面对国内外复杂多变的形势，我们审时度势，主动作为。早着手早准备，积极与开行及其他商业银行沟通合作，完成政府投资50亿元，带动社会投资较快增长。针对去年年初我国南方发生的雨雪冰冻灾害，积极组织农民抢种瓜菜3.89万亩，既支援了灾区，又促进了农民增收。针对四川汶川特大地震灾害，第一时间派出救援队抵达灾区，组织社会捐款8000多万元，充分展示了椰城人民的精神风貌和社会责任。针对国际金融危机的冲击，及时召开旅游、工业、医药、房地产等行业的企业座谈会，出台《关于进一步加快工业发展的若干规定》，修订《关于鼓励发展医药产业的若干规定》和《关于扶持高新技术产业发展的若干规定》，扩大航空、旅行社的奖励范围，积极落实省政府出台的关于扶持房地产、中小企业和非公有制经济发展等9个方面的政策措施，千方百计加大产业培育力度。认真落实中央扩大内需的政策，紧密结合海口发展规划，编制新增投资计划和今后三年滚动投资计划，争取中央扩大内需新增投资2.97亿元，目前已落实今年的政府投资项目资金42亿元，为政府投资项目建设提供了有效的资金保障。

(二)努力壮大优势产业，经济持续健康发展。坚持“一产做优、二产做大、三产做强”，统筹三次产业发展。一是着力发展现代农业。进一步加大支农惠农力度，全市财政安排农林水支出2.74亿元，增长32.4%，落实农业生产补贴6235.08万元。积极发展设施农业，畜牧业、渔业、花卉、水果、瓜菜等特色优势产业产值全面增长。其中畜牧业增幅达到14.1%，水产品出口创汇增长70%。积极发展生态循环农业，与省农业厅联合创办生态循环农业示范区，新建大中型沼气工程70处，服务站13个，新增循环农业基地1.5万亩。

实现农业增加值31.4亿元，增长8.6%。二是积极夯实工业基础。完成狮子岭一期、海马二期、药谷二期的基础设施建设，为企业入园创造了良好条件。支持海宇二期、双成药业、海灵制药等企业的技术改造和扩建，新引进15家企业入园。创建全国知识产权示范市，积极培育发展高新技术企业，新增市级重点实验室5个、创新示范企业5个和工程研发中心5个。除汽车制造业产值下滑外，医药制造、机电制造、食品饮料等行业分别实现25.6%、16.7%、10.2%的快速增长。三是加快发展现代服务业。认真落实《关于加快发展现代服务业的意见》，加大现代物流和旅游业的扶持力度。建成海南花卉交易市场，积极推进马村港一期、海口港二期工程，加快海口中商农产品中心市场前期工作。加大旅游促销力度，积极构建琼北湛江旅游经济圈，成功举办《城市之间》中国区总决赛、中国铁人赛、热气球挑战赛、世界女子高尔夫职业赛等大型赛事活动，组织冬交会、旅游商品交易会、汽车交易会等51场大型会展，旅游经济稳健增长。全年接待过夜游客637.9万人次，增长8.5%，实现旅游总收入60.02亿元，增长8.4%。交通运输、通讯、批发零售、住宿餐饮等服务业快速发展。第三产业完成增加值298.5亿元，增长14.4%。

(三)加大投资力度，重点项目建设取得突出成效。努力创造条件，积极落实贷款融资规模，确保重点续建项目和新开工项目的资金闭合;强化重点项目协调服务和资金调节力度，实行重点项目联席会议、市长办公会和专项督查制度，保续建、保完工，全力推进重点项目建设。绕城高速公路按时实现功能性通车，海口港二期建成试运行，完成东环铁路海口段征地拆迁，海甸岛环岛路、滨江西路等49个续建项目建设进度明显加快，其中22个项目基本完工。20万吨镀锡薄板、30万辆汽车等项目开工建设，灯塔酒店围填海工程、羊山路网工程等一批重点项目完成前期工作。

(四)不断深化重点领域改革，体制活力进一步释放。抓住影响我市发展的重点领域和关键环节，攻坚克难、强力推进。经过各方配合，积极落实，海口综合保税区获国务院批准设立，成为国内开放程度最高、政策最优惠、功能最齐全、手续最简便的特殊监管区域之一。强区扩权取得重大突破，65项行政权项下放各区管理并顺利运作;调整和完善市区分税制财政管理体制，规范市区政府分配关系，调动区级发展的积极性。省政府下放的197项行政管理事项对接迅速，运转正常。认真开展机关效能建设，规范办事行为，简化办事程序，144个行政审批和服务项目进驻政府服务中心，有54项行政事项缩短办事时限。创新公共服务方式，信访、椰城纠风热线、“12345”政府服务热线和“963333”效能投诉热线等工作成效明显，机关工作效率和服务质量明显提高。加强国有资产监管，继续深入推进国有资产管理体制改革，确保国有资产保值增值。投融资体制改革进一步深化，修订《政府投资项目管理暂行规定》，简化投资项目审批流程。创新城市经营方式，举办新浪乐居海口地产项目推介会，新港、玉沙村、司马坡岛等地块成功出让。

(五)加快对外开放步伐，经济活力明显增强。坚持把扩大开放作为保持经济持续发展、推进产业升级的重要举措。利用我市被确定为全国第二批加工贸易梯度转移重点承接地的机会，组团到珠三角地区主动与企业洽谈、与当地政府对接。整合招商资源，创新招商方式，推出招商项目87个，签约项目45个，全年实际利用外商直接投资5.6亿美元，增长11.2%。积极参加海南(香港)经贸周活动，签约项目12个，其中鸿洲新城、垃圾焚烧发电等项目已开工建设。调整出口产品结构，强化出口服务，全年外贸出口总值13.1亿美元，增长15.6%。利用国内外媒体，广泛宣传20年来海口改革开放和现代化建设的巨大成就，成立海外智囊团，举办海口-东盟对话会，成为世界城市与地方政府联合组织亚太地区理事成员，海口的知名度进一步提升。

(六)扎实推进城市化进程，城乡面貌明显改观。围绕建设全国最精最美省会城市目标，坚持科学规划、集约建设和精细化管理，城市规划建设管理跃上新台阶。一是着力提升城市品质。完成海口历史文化名城保护规划、五条老街保护与改造规划、海口火山群国家地质公园总体规划、西海岸新区、江东组团等控规的编制。加快推进美丽沙、新埠岛、世纪海港城、海口湾、西海岸等片区开发。坚持政府主导，让利于民，玉沙村、海甸溪北岸改造取得重大突破。集中人力物力财力，完成主要街道的街景立面改造、小街小巷路面硬化和主要建筑、桥梁的美化亮化。认真开展交通专项整治年活动，加强公共交通基础设施建设，全市300多辆旧式中巴车全部置换成空调大巴。推行文明施工、紧凑施工、规范施工，项目施工管理得到进一步加强。二是加快新农村建设。基础设施建设进一步向农村延伸，农村公路畅通工程提前两年完成“十一五”目标，全市行政村道路基本实现硬化。新建226个农村饮水项目，解决5.1万人的安全饮水问题。继续推进“广播电视村村通”工程，消除40个广播电视“盲点村”。加强农村社会服务体系建设，农业科技110和电子农务覆盖面进一步扩大。全市专业合作社发展到217家，其中3家被评为“全省十佳农民专业合作社”。新建文明生态村170个, 累计建成1069个，占自然村总数的50.3%。三是强化生态环境建设。加快推进白沙门污水处理厂二期、长流污水处理厂建设，继续推进中心区截流并网和府城管网改造工程，新建垃圾转运站6座。完成造林面积4.2万亩，超额完成退塘还林任务;新增城市绿化面积150万平方米，城市人均公共绿地达9.5平方米。我市入围全国最具幸福感城市50强，被评为环境最佳城市，荣获“全国创建文明城市工作先进城市”称号。

(七)强化民生保障，群众得到更多实惠。制定《关于大力改善民生推进基本公共服务均等化的实施意见》，坚持以人为本，着力改善民生。全年财政投入23亿元，增长22.4%;市本级一般预算新增财力的60.5%投向民生，办成了一批事关群众切身利益的实事。投入6.13亿元，全面完成25项为民办实事事项。教育事业进一步发展，教育支出达9.9亿元，增长19.5%，新建扩建学校10所，新增义务教育学位1万个;继续落实“两免一补”政策，免除学杂费、课本费和发放补助共5391万元;加快市旅游职业学校和高级技工学校建设，组建旅游教育集团，职业教育办学水平明显提高;积极支持海南大学“211工程”创建，海口经济学院成功实现专升本。文体事业繁荣发展，建成中国大致坡戏剧家活动基地，并被评为“国家文化产业示范基地”，新建镇级综合文化站15个，全年组织文化活动7845场。公共卫生体系基本建成，强化农村卫生院基础设施建设，社区卫生服务覆盖率达95%，被纳入全国社区卫生服务重点联系市;完善卫生监督体系，疾病控制和医疗服务质量进一步提高;全民医保成果进一步扩大，新型农村合作医疗参合率达98%，城镇居民医疗参保率达97.6%。就业与社会保障体系进一步完善，帮助就业困难人员就业再就业1.24万人，零就业家庭就业扶持政策落实到位;免费培训农村劳动力1.64万人次;对困难群众实行动态补助，压缩4%的市级行政经费，一次性发放给困难群众，城乡困难居民享受低保累计达66.1万人次;大幅提高环卫合同工人工资，月人均工资标准达947元。破解困难群众住房难，投入1.5亿元兴建廉租房2364套，投入17亿元建设安置房、改造棚户区，发放低收入住房困难家庭租房补贴200万元。

(八)加强民主法制建设，社会保持和谐稳定。主动接受人大法律监督和政协民主监督，加强与各民主党派、工商联和无党派人士的沟通联系，定期向市人大、政协通报工作。办理人大代表建议213件，政协提案330件。推行“市长接待日”常态化。进一步完善社会公示与听证制度、新闻发言人制度。提请市人大审议法规议案4件，颁布法规2件，废止规章1件。

深入推进“平安海口”建设，社会治安形势总体稳定，确保了重大活动的圆满安全。积极排查调处各类矛盾纠纷，预防和妥善处置群体性事件。强化应急管理，公共突发事件应急机制逐步健全。落实安全生产责任制，强化监督管理，深化隐患排查治理，安全生产工作在全省综合考核中评为优秀。加大食品、药品等市场监管力度，居民消费安全得到进一步保障。

人口与计划生育工作顺利完成省下达的目标任务，民族、宗教、外事、侨务和对台等工作取得新成绩。人防、统计、拥军优属、防震减灾等工作不断加强。工会、共青团、妇联、科协、红十字会、老龄、关心下一代、残疾人等其他社会事业健康发展。

各位代表，过去的一年，我们经受住了严峻的挑战和考验，保持了经济社会较快发展，成绩来之不易。这是省委、省政府和市委正确领导的结果，是全市人民团结拼搏的结果，是社会各界共同努力的结果。在此，我谨代表市人民政府，向全市人民，向全体人大代表和政协委员，向各民主党派、工商联和社会各界人士，向驻市部队和武警官兵，向所有关心支持海口发展的同志们、朋友们，表示衷心的感谢!

同时，我们也清醒地认识到，海口经济社会发展和政府工作中还存在不少问题。主要表现在：受国际金融危机的影响，我市一些主要经济指标增幅回落，GDP、工业增长没有达到预期目标;汽车、房地产等市场需求萎缩，企业发展面临前所未有的困难，经济下行压力加大;自主创新体系还不健全，产业转型升级任务繁重;影响科学发展的体制机制问题尚未根本解决，改革攻坚任务还很艰巨;公共服务还不适应城市发展的需要，社会管理还存在不少薄弱环节;就业形势较为严峻，居民增收难度加大，食品安全、教育卫生、居民住房、防灾减灾等关系群众切身利益的问题还需要继续加大力度解决，维护社会稳定的任务仍然较重;政府自身的改革和建设有待进一步加强，政府执行力和效能有待进一步提高，一些政府工作人员的服务意识不强、工作作风不实、工作效率不高，腐败现象在有些领域还比较突出。对此，我们将予以高度重视，并采取更加有效的措施加以解决。

二、2009年工作安排

2009年是新中国成立60周年，也是应对国际金融危机、采取一切措施保增长、扩内需、调结构的关键之年。做好今年的工作，意义十分重大。当前，国际金融危机快速蔓延，世界经济增长明显减速，国内经济运行困难急剧增加，今年可能是进入新世纪以来我国经济发展最为困难的一年。尽管我市受外部影响有一个滞后过程，但金融危机的负面影响已经凸显，继续保持又好又快的发展态势，任务异常艰巨。我们必须进一步增强危机感、紧迫感，把困难估计得更充分一些，把应对措施考虑得更周密一些，把各项工作做得更扎实一些。应当看到，这场危机并没有改变我国经济发展的基本态势。党中央、国务院及时准确判断形势，迅速果断作出扩内需保增长的重大决策，为我们提供了克服困难、促进经济增长的有利条件。应当看到，海口处于上升期的发展趋势也没有改变。市第十一次党代会以来，市委作出的一系列重大决策部署，为我们战胜危机，加快发展，赢得了时间，争取了主动，我们完全有基础、有条件、有能力克服当前的困难，实现新的更大发展。应当看到，危机之中蕴含着重大机遇，关键看我们有没有逆势而上、奋力闯关的勇气，有没有变压力为动力、化挑战为机遇的能力。我们坚信，只要全市上下振奋精神，勇往直前，全力以赴，扎实工作，就一定能够战胜各种困难，牢牢把握科学发展的主动权。

今年政府工作的基本思路是：全面贯彻党的十七大和十七届三中全会精神，坚持以邓小平理论和“三个代表”重要思想为指导，深入贯彻落实科学发展观，认真落实中央一系列宏观经济政策，把保增长作为首要任务，把扩大投资上项目作为关键抓手，把扩大消费抓促销作为重要手段，把推进重点领域改革提升开放水平作为根本动力，把改善民生作为出发点和落脚点，把提高行政效能狠抓落实作为重要保障，促进我市经济社会又好又快发展。

今年经济社会发展的主要预期目标是：全市生产总值增长10%，地方一般预算收入增长10%，全社会固定资产投资增长15%，城镇居民人均可支配收入和农民人均纯收入分别增长10%，城镇登记失业率控制在4.5%以内，人口自然增长率控制在9‰以内，万元GDP能耗下降2.5个百分点。

根据上述目标，我们要重点抓好以下五个方面的工作：

(一)扩大有效需求，切实增强经济发展后劲

坚决贯彻落实中央和省扩大内需的各项政策措施，进一步增强投资和消费对经济增长的拉动作用。

千方百计加大投资力度。把狠抓项目、扩大投资作为保增长的主攻方向和着力点，创造条件，能上则上，能快则快，确保实现投资预期目标。今年，全社会固定资产投资力争完成252亿元。一是扩大政府投资。今年市政府固定资产投资要翻一番，优先重点安排民生、重大基础设施和产业配套项目，引导和带动社会投资。发挥融资平台作用，用足用好国务院《关于当前金融促进经济发展的若干意见》，加强与金融机构的项目对接，争取更多的信贷支持。二是大力推进项目建设。全力推进8大类94个重点项目建设，实行跟踪服务、严格督办，切实解决征地拆迁、市政配套等问题，优化项目施工环境，确保一批续建项目早日建成投产，一批新项目早日开工建设，确保重点项目建设推进力度。三是扩大社会投资。降低社会投资门槛，积极探索新的投融资模式和渠道，引导民间资本投向政府鼓励项目和符合国家产业政策的领域，广泛参与民生工程、公共设施和生态环境建设。四是加快项目储备。编制100个产业项目、100个基础设施项目、100个物流项目、100个旅游地产项目、100个社会发展项目，强化项目的包装孵化，做好项目与国家政策的对接，积极争取更多的项目纳入中央和省的投资盘子。

千方百计引进岛外需求。引进岛外需求是我市保增长的重要手段。要采取多种方式更多地引进岛外需求。一是策划组织大型推介活动。我市的度假休闲旅游、房地产和热带农产品岛外市场空间较大，必须抓好促销。今年安排1600万元促销经费，到销售热点地区和有潜在需求地区，有针对性地开展大型促销和推介活动，拓展岛外市场。二是创新促销方式。实行政府搭台、企业唱戏的方式，整合旅游、房地产和农产品营销力量，开展经常性促销活动。综合利用各种媒体宣传促销，搭建旅游房地产促销网络平台，扩大网上交易。三是大力发展会展经济。围绕旅游、房地产和农产品，策划一批特色鲜明、吸引力强的大型会议会展、节庆娱乐、文体赛事活动，采取更加积极的扶持政策，争取更多的区域性、全国性、国际性会议和活动落户海口。

千方百计拉动岛内消费。坚持把挖掘全省的消费需求作为扩大内需的重要着力点。一是增强居民消费能力。多渠道增加居民特别是中低收入居民收入，完善社会保障体系，稳定收入预期，确保即期消费快速增长。二是大力培育消费热点。积极策划和举办多种形式的商品促销活动，不断扩大游客和其他市县居民在海口消费;扩大信用消费，着力培育汽车、旅游、通讯、文化、健身、信息等新兴消费热点。三是促进住房消费。认真落实中央和省关于促进房地产市场稳定健康发展的政策措施，支持和鼓励普通自住住房和改善型住房消费，加快发展二手房市场和住房租赁市场，促进房地产业持续健康发展。四是扩大农村消费市场。继续实施“万村千乡”市场工程，加快城乡消费品、生产资料和农产品流通网络建设;开展家电下乡、农机下乡活动，兑现农民购买家电、农机具的补贴，努力扩大农村居民消费。

(二)深入推进产业结构调整，促进经济发展方式转变

坚持把保增长、扩内需、调结构有机结合起来，提高自主创新能力，加快经济结构战略性调整，进一步增强发展的协调性和可持续性。

加快发展现代农业。保增长的基础支撑在农业，扩内需的最大潜力在农村，保民生的重点难点在农民。要拿出真招实策，舍得增加投入，夯实农业基础，增加农民收入。今年财政对“三农”投入1.145亿元，增长79.8%。一是加快调整农业内部结构。进一步优化农业产业布局，重点加快海榆东线、海文高速生态循环农业示范区，西秀镇农业示范园和羊山特色果蔬产业带建设，大力发展热带高效农业、畜牧业、农副产品加工业，提高农产品附加值，积极推进农业产业化经营。继续扩大设施农业规模，投入6374万元，带动社会资金，新建13个生态养殖小区、13个大中型沼气池和50个农村沼气服务网点，发展1.3万亩生态循环农业基地和2000亩的花卉产业基地，进一步扩大设施农业在农业中的比重。二是增强科技对农业的支撑。强化农业实用技术培训，推广良种良苗，扩大延伸农业科技110服务网络，抓好农产品检测检验体系和农产品追溯制度建设，确保农产品质量安全合格率达到96%以上，扩大农产品、水产品出岛出口和进宾馆进超市的规模。三是加强农村基础设施建设。推进病险水库除险加固、大型灌区续建配套和节水改造，全面解决农村饮水不安全问题。继续实施农村畅通工程，新建农村公路512条、1124公里。加快农村环卫基础设施建设，切实改变农村的环境卫生状况。加快推进农村文化基础设施建设，配备23个镇综合文化站的设施，丰富群众文化生活。四是落实强农惠农政策。健全农业投入保障机制，继续提高良种、农机具购置、农资综合直补等补贴标准，扩大政策性农业保险险种和覆盖范围。

加大工业发展扶持力度。我们要充分估计金融危机对工业的冲击，动员全市上下聚焦工业、服务工业、发展工业，形成调动各种要素促进工业发展的局面。一是实施支柱产业振兴计划。抓住国家实施产业振兴规划的机遇，抓紧编制我市支柱产业振兴实施计划，用足用活国家和省鼓励企业发展的优惠政策，加快落实我市已出台的《关于进一步加快工业发展的若干规定》、《关于鼓励发展医药产业的若干规定》、《关于扶持高新技术产业发展的若干规定》和《关于加快发展现代服务业的意见》等扶持产业发展的措施，安排2亿元产业扶持资金，完善中小企业担保体系，对高新技术企业和符合条件的中小企业采取担保、贴息等扶持政策，鼓励企业技术进步和产品转型升级。二是加快工业园区建设。优化海口药谷、海马工业园、狮子岭工业园配套服务功能，启动桂林洋农副产品加工区、云龙产业园区建设，切实推进20万吨镀锡薄板、30万箱红塔山卷烟厂扩建，加快30万辆汽车项目建设，促进工业集中布局、集约发展。三是着力培育高新技术产业。推进知识产权示范市建设，制定高新技术产业发展规划，继续支持重点实验室、创新示范企业和工程研发中心建设，鼓励企业产品研发和技术创新;积极争取省产业发展引导资金支持，充分发挥市高新技术产业专项资金和科技风险投资的引导作用，组织实施一批高新技术产业化示范项目，促进电子信息、新能源、新材料、生物制药等产业加快发展。四是尽力帮扶企业渡过难关。要统一认识，帮企业，就是保增长、保就业、保稳定、保后劲。综合运用各类金融工具，加大对中小企业的支持，着力解决企业融资难问题。切实加强和改进对企业的服务，建立政府部门与各类企业经常性沟通联系制度，及时了解、把握、分析企业发展情况，适时研究采取力度更大的政策措施，帮助企业克服困难。清理和规范收费、年检等事项，对全市企业的行政性收费和年检事项进行全面清理，不合理的坚决废除，切实为企业减负。

大力发展现代服务业。加大《关于加快发展现代服务业的意见》的实施力度，依托省会中心城市优势，积极发展以旅游为龙头的现代服务业，扩张总量、优化结构，着力提高服务业在国民经济中的比重。一是进一步提高旅游业的总体效益。抓住中国生态旅游年和国民休闲计划的机遇，强化旅游宣传促销，境外重点推介高尔夫游、热带滨海温泉度假游等产品，国内重点推介商务游、生态游、乡村游等产品，全力拓展旅游客源市场。抓紧编制我市国际旅游岛建设行动计划，推进邮轮游艇码头、免税商城、雷琼世界地质公园海口火山群博物馆、海南游客到访中心等重点旅游项目建设，积极吸引国际著名酒店管理集团落户我市，促进旅游基础设施上档次、旅游服务上水平。继续打造琼北湛江区域旅游经济圈，推出旅游精品线路，努力推进“住在海口、玩在琼北”的旅游目的地建设。二是积极发展现代物流业。继续抓好新海滚装客运港、海口港二期、马村港一期建设，加快推进粮食物流、新海物流园区建设，支持海南钢材、中商农产品、海南花卉交易市场等特色中心市场建设，促进和提高物流业规模化和集约化发展，增强区域物流中心的辐射能力。三是扶持发展其他服务业。进一步落实服务业发展的优惠政策，培育发展金融保险、信息咨询、文化创意、影视娱乐等新兴服务业，改善提升餐饮、商贸等传统服务业，切实推进医疗卫生、教育培训、文化体育等服务业。

(三)纵深推进改革开放，增强经济发展活力

进一步解放思想，发扬敢闯敢干、敢为人先的精神，大胆探索，不断加大改革力度，提升对外开放水平，为保增长、扩内需、调结构提供强大动力。

深化各项改革。坚持社会主义市场经济改革方向，积极推进重点领域和关键环节的改革创新，进一步完善保障科学发展的体制机制。一是推进农村综合改革。继续推进乡镇政府职能转变，增强乡镇政府的社会管理和公共服务职能;开展土地承包经营权流转试点，发展多种形式的适度规模经营;全面推进集体林权制度改革，促进林业产业发展。二是深入推进行政管理体制改革。结合新一轮的机构改革，积极探索大部门制，创新行政管理体制，加快政府职能转变;结合省下放的197项行政权项的规范运作，继续加大强区扩权的实施力度，增强下放权项的协调性和实效性，切实提高区级自主发展经济的能力。三是深化社会事业改革。积极推进医药卫生体制改革，着力提高医疗卫生的管理能力和服务水平，为群众提供安全有效、方便价廉的医疗卫生服务。推动社会保障、文化教育、事业单位等各项改革。四是积极促进社会组织发展。认真落实市委《关于进一步转变政府职能促进社会组织发展的决定》，有计划地将一些职能部门的管理权项向行业协会和社会组织下放，发挥社会组织在管理社会事务中的主导作用。

拓展开放领域。准确把握金融危机引发的国际产业资本流动的新趋势和国际市场格局调整的新变化，采取更加灵活的开放措施，推动开放型经济转型升级。一是提高利用外资水平。发挥加工贸易产业转移重点承接地优势，精心论证策划一批大项目、好项目对外招商，突出抓好先进制造业、高端服务业、高新技术、总部经济、基础设施等领域的招商引资。跟踪抓好在谈、在批、在建项目，办好重大招商活动，完善招商项目推进机制。二是全面优化开放环境。保护外资企业合法权益，强化部门联动服务机制，排除影响发展的制约和障碍。帮助企业开拓国际市场和化解贸易磨擦，完善大通关体系，进一步降低通关成本，确保出口退税“应退尽退”。三是加强开放载体建设。完善与澄迈县合力推进海口综合保税区建设的联动机制，定期召开协调会，尽快完成征地拆迁和基础设施建设，开展全方位的招商选资，争取一年内封关预验收，使其成为我市乃至琼北发展的新动力。

(四)打造中心城市板块，加快温馨宜居城市建设

围绕建设全国最精最美省会城市的目标，以规划为先导，以片区开发为突破口，把必要财力集中投入到新区开发和旧城改造上，进一步完善省会中心城市功能，提高城市综合承载能力。

加快新片区建设和旧城改造步伐。集中力量加快推进长流新区建设，以长流起步区路网、第二办公区、国际会展中心的建设为突破口，带动长流片区的整体开发，争取两年出形象、三年成规模。加快美丽沙、新埠岛、世纪海港城、白沙门生态公园、海口湾游艇泊位码头建设;启动江东片区、金沙湾片区的开发，打造特色鲜明的滨海生态新城区。加快旧城改造，坚持“政府主导、让利于民、补偿合理、安置到位”的原则，整合资源、集中资金，加快海甸溪北岸、朱云路片区改造，启动骑楼建筑历史文化街区的保护和综合整治，让旧城区和棚户区的人民共享城市发展成果。进一步加强中心镇建设，发挥市区两级融资平台的作用，吸纳社会资金参与中心镇的基础设施建设;支持有条件的中心镇兴建建筑装饰材料、花卉苗木、农产品等专业批发市场，规划引导农家乐和旅游产品加工业的发展，促进中心镇二、三产业的发展。

完善省会中心城市功能。继续推进路网建设，完善以八纵四横主干道为骨架，次干道、支线路合理分布的城市道路网路。坚持“以港兴市”战略，加快推进马村、秀英、新港、新海港的建设，提升海口港主枢纽港地位。结合东环快速铁路，规划建设与城市路网的连接站点。加快推进美兰机场第二跑道建设，启动美兰机场临空产业园区建设。坚持公共交通优先发展，加快公共乘换枢纽建设，继续在快速路和主路上设立公交客运走廊和站点，逐步完成公交在城市客运交通系统中的主导地位。完成白沙门污水处理厂二期、长流污水处理厂、龙塘饮用水源点环境保护工程建设，加快中心区和新片区污水截流并网工程，进一步提高城市污水处理率。

提高城市管理水平。推进城市社区建设，按照人性化、规范化、生态化的要求，打造一批体现城市管理水平和文明程度的样板社区。搞好市区环境综合整治，继续推进违法建筑专项整治，遏制城乡新增违法建筑，进一步完善长效管理机制。切实加强生态环境保护，加快“生态海口”、“温馨宜居”工程建设，使环境更加优美，生活更加方便，道路更加畅通，城市魅力更加凸显，市民生活更加幸福。

(五)全面发展社会事业，切实保障和改善民生

保障和改善民生，是政府义不容辞的职责。当前，国内外经济形势严峻，必然给民生工作带来诸多困难。越是在困难的时候，越是要高度关注民生。我们将始终牢记为人民服务的宗旨，加大民生投入，改善群众生活，保障群众利益，努力开创全市人民安居乐业、共享和谐的新局面。

确保就业形势基本稳定。把推动就业再就业工作放在更加突出的位置，今年新增就业人员2.8万人。实施更加积极的就业政策，启动新一轮小额贷款担保工作，支持更多的劳动者成为创业者。努力稳定现有就业岗位，实施与就业挂钩的企业发展支持政策，引导企业采取在岗培训等多种措施不裁员或少裁员。努力扩大旅游职业学校、高级技工学校的招生规模，新增中等职业学位3000个，让更多中学、大中专毕业生继续就学深造，缓解就业压力。

投入2580万元，完善城乡就业公共服务和救助体系，加强对下岗、失业人员的就业技能培训，健全城镇零就业家庭、农村低保家庭等就业困难群体的就业援助长效机制。

努力办好人民满意的教育。今年财政安排教育专项支出2.26亿元，增长83.3%。要继续开展教育质量年活动，进一步推进规范化学校建设，大力实施校长、教师素质提高工程，完善城乡教师交流机制，促进基础教育均衡发展。安排2.05亿元，加快琼山中学、琼山华侨中学、琼山一小等学校扩建改造工程，新增1万个学位，基本解决府城地区学位紧缺问题。安排1.53亿元，落实义务教育阶段“两免一补”助学工程，扩大城乡学生受益面，保障困难家庭、进城务工人员子女平等接受义务教育。推进高中阶段教育结构调整。加快完善桂林洋大学园区的配套设施建设，积极支持海南大学“211工程”创建，提高海口经济学院、海南职业技术学院的办学水平。

加强公共卫生体系建设。今年财政安排医疗卫生专项支出7112万元，增长39.6%。完成市医院综合大楼、中医院、疾病控制中心、“120”急救中心、乡镇卫生院的建设。进一步完善卫生应急体系，建立卫生长效应急机制。安排830万元，加快农村和社区卫生服务体系建设，确保农村卫生室和社区卫生服务网点覆盖率都达到100%。全面加强人口和计划生育工作，进一步稳定低生育水平。

进一步提高社会保障水平。今年财政安排社会保障专项支出9813万元，增长180.1%。要巩固和扩大全民医保的覆盖面，确保新型农村合作医疗参合率和城镇居民基本医疗参保率稳定在95%以上，提高人均参保补助标准。完善最低生活保障标准的动态增长机制和物价补贴机制，继续提高离退休人员基本养老金标准，切实保障城乡困难家庭、离退休职工的基本生活水平不下降。安排1000万元，新建、扩建4个敬老院和4个“五保之家”，不断提高农村五保户和城镇“三无”人员的集中供养率。

提高住房保障水平。推进保障性安居工程，加大廉租住房保障力度，加快经济适用房建设步伐，探索建立政府限价房、农民工住房等制度，多渠道解决城市中低收入群众住房困难。今年安排2.98亿元，建设2575套廉租住房。采取货币补贴与实物配租相结合的方式，对低收入家庭实现廉租住房应保尽保。

促进社会和谐稳定。推动城市报警与监控系统工程建设，认真落实社会治安综合治理各项措施，进一步抓好禁毒工作，严厉打击各类违法犯罪活动。加大矛盾纠纷排查和重信重访专项治理力度，有效化解各类矛盾，积极预防和妥善处置群体性事件。扎实开展安全生产年活动，强化安全生产监管，加大执法检查和综合整治力度，加快应急救援体系建设，坚决遏制重特大事故发生。加强食品药品监管，严厉打击违法违规生产经营行为，维护人民群众生命财产安全。

深入开展文明城市创建工作，加强社会主义核心价值体系建设，完善学校、家庭、社会相结合的未成年人教育网络。高标准做好双拥工作，促进军地军民和谐。发挥工会、共青团、妇联等人民团体在创建文明城市中的作用，加强老龄工作，推进妇女儿童和残疾人事业的发展。大力开展爱国卫生运动。继续做好外事侨务、民族宗教、档案史志、地震等工作。

三、切实加强政府自身建设

实现保增长、扩内需、调结构的目标任务，政府肩负着重要责任。我们一定要在市委的领导下，切实加强政府自身建设，努力创造良好的发展环境，促进经济社会又好又快发展，决不辜负党和人民的重托。

进一步提高领导科学发展的能力。深入开展学习实践科学发展观活动，深刻领会和准确把握科学发展观的丰富内涵，学习新知识、研究新情况、解决新问题，进一步增强各级领导干部和公务员贯彻落实科学发展观的自觉性和坚定性。正确处理当前与长远的关系，坚持一切从实际出发，强化对经济运行的调控，不断提高政府的执行力，真正把保增长、扩内需、调结构的任务落到实处。严格按照法定权限和程序行使权力、履行职责，自觉接受市人大及其常委会的法律监督和市政协的民主监督，主动接受舆论监督和社会监督;继续办好“椰城纠风”热线和“12345”政府服务热线，完善“市长接待日”制度，不断提高依法行政水平。

进一步提高行政效能。认真开展以“帮助扶持企业发展、帮助推进项目建设，促进民生改善、促进经济增长”为主要内容的“执行年”活动，深化机关效能建设。推进政府工作项目化，各项工作都要明确目标，制定实施方案，实行量化管理，确保省委、省政府交办的重点工作、责任状事项和市委的决策部署按时完成。深化行政审批制度改革，理顺审批职能，减少审批事项，改进审批流程，实行并联并行审批，审批时限再压缩30%以上;加快电子审批网络和电子监察系统建设，行政许可项目在线审批达50%以上;完善重点项目联席会议制度和跟踪督办制度，建立重点项目建设绿色通道，实行一个部门牵头、一个窗口受理、一套数据审批、一次现场勘查、一次集中会审，实现政府服务大提速。强化监督检查，保证政令畅通，对执行不力的责任人和责任单位坚决实施行政问责。建立完善绩效考评机制，激发队伍活力，变“要我干”为“我要干”，以服务企业、服务项目、服务市民的实绩检验政府的行政效能。

进一步加强勤政廉政建设。坚持把以人为本的执政理念体现在各项工作中，落实到经济社会发展的各个方面。尊重群众主体地位，发挥群众首创精神，坚持问政于民、问需于民、问计于民，继续实施为民办实事工程，扎扎实实为基层、企业和群众排忧解难。大力弘扬埋头苦干、务实创新的工作作风，要开短会、发短文、多调研，坚决取消各种没有实际内容的会议和活动。牢固树立艰苦奋斗、过紧日子的思想，今年公务购车用车、会议、公务接待和机关工作人员出国出境经费支出零增长，将有限的财力更多地投向社会民生事业。坚决贯彻执行廉洁从政的各项规定，认真落实党风廉政建设责任制，利用我市少数干部的腐败典型案例，开展警示教育，推进反腐倡廉制度建设。加大违法违纪案件查处力度，坚决纠正损害群众利益的不正之风。加强对建设工程、土地出让、产权交易和政府采购等重点领域的监管，形成公开、公平、公正的运行机制，努力建设节俭、清廉、对人民负责的政府。

各位代表，海口发展正处在一个关键时期，困难和挑战考验着我们，责任和使命激励着我们。让我们更加紧密地团结在以胡锦涛同志为总书记的党中央周围，在省委、省政府和市委的坚强领导下，坚定信心，把握机遇，团结奋斗，扎实工作，为建设繁荣富裕、环境优良、社会和谐的海口而努力奋斗，以优异的成绩向新中国成立60周年献礼!
